# RhoA/rho kinase pathway activation in age‐associated endothelial cell dysfunction and thrombosis

**DOI:** 10.1111/jcmm.18153

**Published:** 2024-04-03

**Authors:** Iván Palomo, Sergio Wehinger, Vicente Andrés, Francisco J. García‐García, Eduardo Fuentes

**Affiliations:** ^1^ Department of Clinical Biochemistry and Immunohematology, Faculty of Health Sciences, Medical Technology School, Thrombosis and Healthy Aging Research Center Universidad de Talca Talca Chile; ^2^ Centro Nacional de Investigaciones Cardiovasculares (CNIC) Madrid Spain; ^3^ Centro de Investigación Biomédica en Red en Enfermedades Cardiovasculares (CIBERCV) Madrid Spain; ^4^ Department of Geriatric Medicine Hospital Universitario de Toledo, Instituto de Investigación de Castilla La Mancha (IDISCAM), CIBERFES (ISCIII) Toledo Spain

**Keywords:** ageing, cardiovascular disease, endothelial dysfunction, frailty, RhoA/rho kinase pathway, thrombosis

## Abstract

The small GTPase RhoA and the downstream Rho kinase (ROCK) regulate several cell functions and pathological processes in the vascular system that contribute to the age‐dependent risk of cardiovascular disease, including endothelial dysfunction, excessive permeability, inflammation, impaired angiogenesis, abnormal vasoconstriction, decreased nitric oxide production and apoptosis. Frailty is a loss of physiological reserve and adaptive capacity with advanced age and is accompanied by a pro‐inflammatory and pro‐oxidative state that promotes vascular dysfunction and thrombosis. This review summarises the role of the RhoA/Rho kinase signalling pathway in endothelial dysfunction, the acquisition of the pro‐thrombotic state and vascular ageing. We also discuss the possible role of RhoA/Rho kinase signalling as a promising therapeutic target for the prevention and treatment of age‐related cardiovascular disease.

## 1 INTRODUCTION

The progressive ageing of the world population is resulting in a higher prevalence of age‐related disorders, including frailty and cardiovascular disease (CVD).^1^, ^2^ In recent decades, frailty has emerged as an underlying health condition that largely explains the most concerning health problems in older adults, which include hospitalisation, disability, falls and a high mortality risk.^3^ Initially confined to the elderly, frailty now increasingly affects younger individuals, in whom it shows a strong association with multimorbidity and, as in the elderly population, is an important risk factor for harmful events.^4^ These findings position frailty as an important focus of public health efforts.

Frailty syndrome describes a clinically recognisable complex state in older adults who exhibit increased vulnerability and dependency caused by gradual and progressive abnormal functioning of multiple organ systems.^5^ In recent years, there has been a growing interest in the two‐way relationship between frailty syndrome and CVD.^6^, ^7^ The disability precipitated by frailty contributes to the appearance of CVD in the elderly; conversely, clinical and subclinical vascular disease are risk factors for frailty.^6^, ^7^ This interaction between vascular alterations and frailty appears to operate from early stages, with the risk of frailty showing an association with elevated levels of the endothelial dysfunction marker ADMA (asymmetric dimethylarginine) in individuals without atherosclerotic disease.^8^ ADMA is a metabolic byproduct of the continuous metabolism of proteins in the organism and acts as a competitive inhibitor of the enzyme eNOS. Therefore, it is commonly used as endogenous marker of endothelial dysfunction.^9^ This association suggest a relevant role of vascular system dysfunction as one of the main mechanisms leading to frailty. Progression from endothelial dysfunction to thrombus formation is dependent on platelet adhesion, since the alteration of endothelium‐platelet interaction is a well‐recognized contributor to prothrombotic states.^10^ However, there has been little research into the relationship between endothelial dysfunction in frail elderly people and the early actions of platelets before thrombotic cardiovascular events such as myocardial infarction and stroke. Identification of the mechanisms underlying endothelial and/or platelet alterations that lead to cardiovascular events in frailty could contribute to measures to detect, treat and prevent CVD risk in frail elderly people.

Rho GTPases regulate multiple cellular processes, such as cytoskeletal reorganisation, cell migration, microtubule dynamics, signal transduction and gene expression,^11^ and there is a large body of evidence that activation of the RhoA/Rho kinase pathway plays a major role in various forms of CVD and acts as a convergent node in the pathogenesis of endothelial dysfunction.^12^, ^13^, ^14^ Endothelial injury in rats and RhoA/Rho kinase activation in stress‐treated EC cultures are associated with increased levels of biomarkers found in frail human adults, such as ADMA, endothelin 1 and 8‐isoprostane, suggesting a possible implication of the RhoA/Rho kinase pathway in the endothelial dysfunction occurring in frailty.^15^, ^16^, ^17^ However, studies to date have not specifically addressed the direct contribution of RhoA/Rho kinase pathway activation to frailty syndrome or its role in the increased thrombosis risk in frail older adults.

This review summarises the role of RhoA/Rho kinase signalling in endothelial dysfunction, thrombosis and vascular aging and its possible role as a promising therapeutic target for the prevention and treatment of CVD in the elderly population.

## 2 POPULATION AGING

**FIGURE 1:**
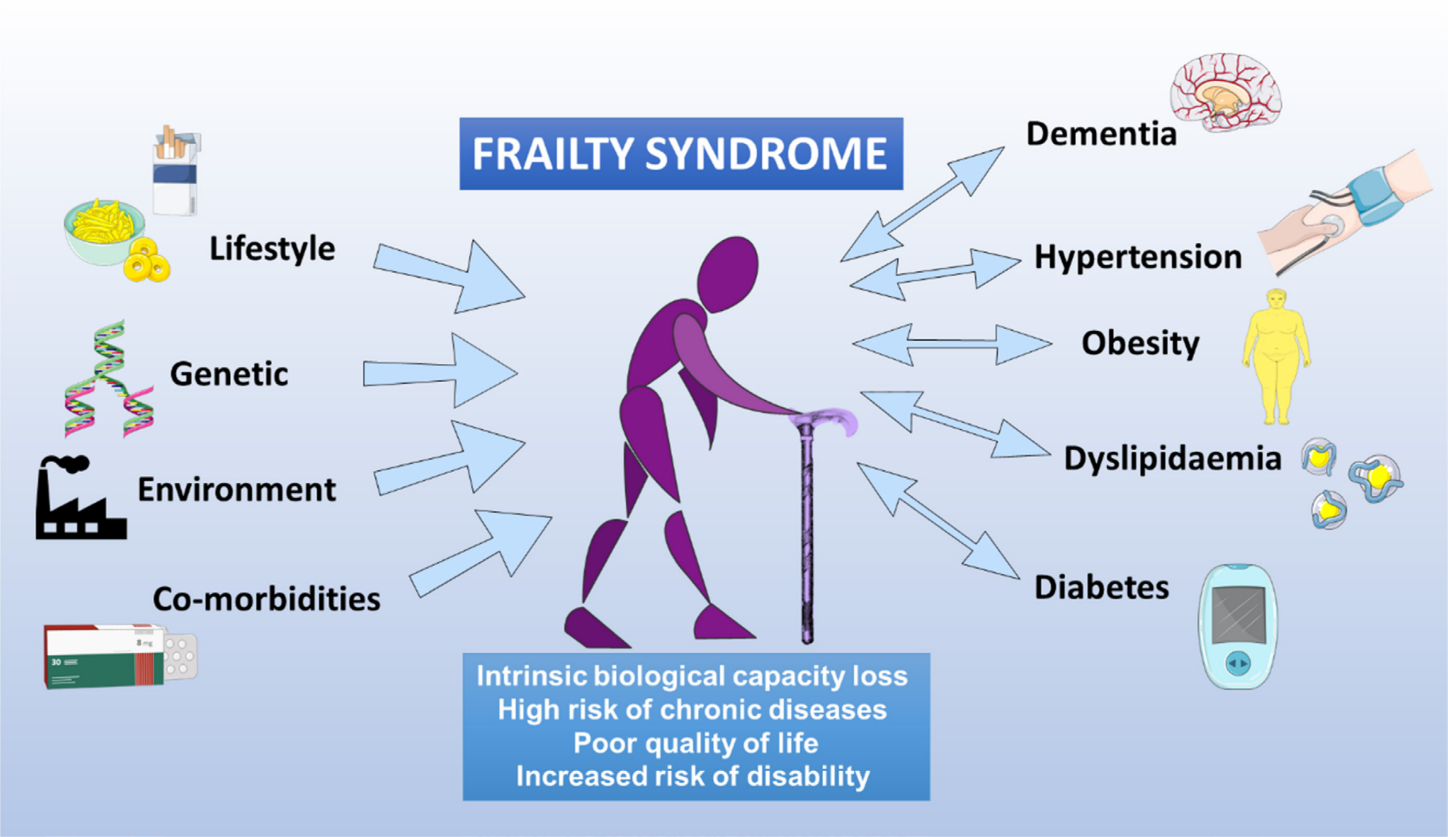
Frailty syndrome. Frailty is characterised by a significant decrease among elderly people in their biological capacity to confront the challenges of daily life. The multiple factors that can trigger the appearance of frailty include lifestyle, genetic background, environmental quality and the existence of co‐morbidities. Frailty status is an important risk factor for chronic diseases associated with old age, which themselves promote the development of frailty.

The world population is ageing rapidly due to increased life expectancy and decreased fertility rates.^18^, ^19^ World Health Organization figures show that the world population older than 60 years more than doubled from 382 million in 1980 to 962 million in 2017 (11% of the total world population). This figure is projected to double again by 2050 to reach nearly 2.1 billion, representing an estimated 22% of the total population.^1^, ^20^ This trend presents an immense challenge for health care systems because aging, particularly unhealthy aging, entails the loss of intrinsic biological capacity and an increased risk of developing chronic diseases (including dementia, diabetes, hypertension, obesity and kidney disease) and the combination of frailty and dependency characteristic of frailty syndrome^21^, ^22^ (Figure 1). This compendium of ageing‐related changes results in significant loss of life quality and social activity and a high risk of disability.^22^, ^23^, ^24^, ^25^

## 3 FRAILTY SYNDROME IN OLDER ADULTS

Older adults are a highly heterogeneous group, with different genetic, biological and environmental backgrounds and life histories. Consequently, older adults of the same chronological age can have different biological ages.^22^, ^26^ Personal biological age can be estimated from a person's frailty index, which is a sensitive predictor of survival.^27^, ^28^, ^29^ *The* frailty index is a continuous grading of age‐related deficit accumulation that provides a threshold above which the loss of physiological reserve and adaptation capacity manifests as functional deterioration.^30^, ^31^ Since several of the biological processes of ageing are modifiable, identifying and preventing frailty syndrome in older people is essential for extending healthy lifespan.^32^, ^33^ The term frailty thus describes a subset of older adults who appear weaker and more vulnerable than their age‐matched counterparts, despite having similar comorbidities and demographic characteristics.^34^

The most widely used measure of frailty is that proposed by Linda Fried et al.^35^ for a population older than 65 years and defined as the frailty phenotype. Older adults with frailty syndrome have a markedly elevated risk of falls, hospitalisation and death, with frailty being one of the best predictors of worsening mobility or difficulty in performing activities of daily living (ADL disabilities).^22^ Recently, a frailty syndrome diagnosis and clinical follow‐up scale has been developed, called “frailty trait scale”. This scale is based on Fried's frailty phenotype but expands its range of evaluation to include all domains of the syndrome, providing a helpful tool for research and clinical practice.^36^, ^37^

In an observational study, frailty syndrome was present in 25%–50% of men and women 65 years and older with chronic diseases such as hypertension, obesity, dyslipidemia, dementia and diabetes.^38^ Frailty syndrome has been proposed as a prognostic and risk stratification factor for coronary heart disease and slow gait speed (an increased time that a person takes to walk a specified distance on a surface over a short distance), showing a higher association than other parameters (OR: 3.8).^35^, ^39^, ^40^, ^41^ In a systematic review of 9 studies encompassing 54,250 patients older than 60 years with severe coronary artery disease or heart failure, the prevalence of frailty was 50%–54%, and this was associated with an OR of 1.6 to 4.0 for all‐cause mortality over a mean weighted follow‐up of 6.2 years.^42^

A meta‐analysis of frailty syndrome among persons older than 60 years in Latin America and the Caribbean detected a mean prevalence of 19.6% (95%CI 15.4–24.3), with values ranging from 7.7% to 42.6%.^43^ The highest prevalence in this range (42.6%) was detected in a cohort of 1301 older adults from Santiago, Chile, analysed in 2008.^44^ However, other studies in Chilean adults older than 60 years in the Maule and Santiago Metropolitan regions reported prevalence values of 24.6% and 13.9%, respectively,^45^, ^46^ with the latter value similar to the Latin American average. Nevertheless, these values are much higher than those observed in European countries such as Spain (8.4%) and Germany (2.8%).^2^, ^47^ The latter could be related to the socioeconomic and quality of life differences between Latin America and Europe, since frailty is related with cognitive functioning, educational level and nutritional status in older adults.

## 4 FRAILTY SYNDROME AND VASCULAR INJURY

Ageing is associated with a series of structural and molecular changes in the vasculature, independently of other cardiovascular risk factors.^48^ These changes involve a disruption of the balance between vasoconstrictor and vasodilator molecules, which leads to a decrease in nitric oxide (NO) availability and an increase in the production of reactive oxygen species (ROS) associated with mitochondrial dysfunction.^49^, ^50^, ^51^, ^52^, ^53^ There is also strong experimental and clinical evidence that aging is accompanied by low‐grade inflammation, termed inflammaging.^54^ Inflammaging has been detected in numerous mouse models and in older human adults and is characterized by increased circulating levels of pro‐inflammatory interleukins such as interleukin (IL)‐6 and IL‐1β.^55^, ^56^

Frailty syndrome has been widely characterised as a pro‐inflammatory and oxidative phenomenon that promotes vascular dysfunction,^8^, ^51^, ^57^, ^58^, ^59^, ^60^, ^61^, ^62^ triggering platelet activation and adhesion to activated endothelium through increased cytokine release and expression of adhesion molecules.^63^ However, knowledge is limited about molecular and cellular mechanisms through which frail adults develop endothelial dysfunction and its potential role in platelet activation. Next, we present the main findings found in frail older people.

### 4.1 Platelet activation and thrombosis risk

Thrombosis risk increases significantly with age, and thrombosis is a common risk factor of morbidity and mortality in frail older people aged 65 years and older,^64^, ^65^, ^66^ who have an elevated risk of thrombotic events (OR: 1.79, 95%CI 1.02–3.13).^66^ In the initial stage of atherosclerosis, platelets adhere to the damaged endothelium and secrete molecules that amplify endothelial dysfunction, such as inflammatory mediators, chemokines, TNF superfamily factors and adhesion proteins.^67^, ^68^ In the final stage, after plaque rupture, platelets adhere to the damaged endothelium and aggregate to form a thrombus, blocking tissue irrigation and oxygenation.^69^ The pathological mechanisms underlying the elevated thrombosis risk in frail older adults are not fully understood.^34^, ^70^

Evidence acquired in recent years links frailty syndrome to abnormalities in platelets that trigger their activation.^71^, ^72^, ^73^ Compared with healthy young people and non‐frail older adults, frail patients have higher levels of platelet activation, demonstrated by increased P‐selectin expression^58^, ^74^ and a significantly higher increase in the binding of PAC‐1 (active glycoprotein [GP] IIb/IIIa) upon stimulation with 1 μM adenosine diphosphate (ADP).^72^

The diagnosis of frailty is normally based on specific clinical criteria, and there is a clear need to identify and validate robust biomarkers for this condition.^34^ Recent work by our group showed that frail adults older than 64 years have higher levels of platelet aggregation and activation (indexed by P‐selectin exposure and activated GPIIb/IIIa) than age‐matched non‐frail older adults, as well as higher plasma levels of thromboxane B2, 8‐isoprostane and growth differentiation factor (GDF)‐15 (a biomarker of mitochondrial dysfunction and cellular senescence).^75^ We have also shown that frail older adults have higher concentrations of platelet‐derived microvesicles (P2RY12^+^/AV^+^).^76^ This increased platelet activity in frail adults is associated with a decreased response to the antiplatelet drug acetylsalicylic acid.^77^, ^78^

Moreover, there is increasing evidence that platelets play a key role in the pathogenesis of vascular injury. Circulating activated platelets secrete a wide variety of molecules that favour the onset and progression of endothelial damage, such as cytokines, chemokines, TNF superfamily ligands, metalloproteinases, and other mediators.^10^, ^79^

### 4.2 Endothelial dysfunction

Endothelial dysfunction is an important contributor to atherosclerosis^80^ and its thrombotic complications.^67^, ^81^, ^82^ Endothelial cells (ECs) are a principal target through which ageing promotes vascular deterioration.^83^ Frailty has been linked to endothelial dysfunction,^8^ evidenced by increases in key markers: adhesion intercellular molecule 1 (ICAM‐1),^78^, ^84^ endothelin‐1,^85^ Von Willebrand factor (VWF),^86^ thrombomodulin,^87^ ADMA,^8^ IL‐6^88^ and c‐reactive protein.^89^ ICAM‐1 and VWF favour platelet adhesion, whereas endothelin‐1 induces platelet activation in patients with coronary heart disease or myocardial infarction.^90^ As previously mentioned, ADMA is an endogenous inhibitor of NO synthase (NOS) and an independent cardiovascular risk factor,^91^, ^92^ and frailty has been associated with increasing levels of ADMA in subjects without atherosclerotic disease.^8^ Likewise, IL‐6 and C‐reactive protein contribute to the prothrombotic state,^93^ and IL‐6 has been positively associated with frailty in men.^94^ Besides, C‐reactive protein increases with age, and increased plasma levels have been proposed as a biological component of frailty.^95^

The term *endothelial dysfunction* encompasses several forms of abnormal endothelial activity, including impaired production of messenger molecules and increased expression of proinflammatory molecules.^96^ A hallmark of endothelial dysfunction is decreased NO availability, due either to enhanced inactivation or to reduced synthesis.^97^ One of the most important contributors to endothelial dysfunction is oxidative stress, which is characterised by an imbalance between the generation of endogenous ROS and antioxidant defence mechanisms.^98^ In fact, frailty and pre‐frailty seem to be associated with higher oxidative stress,^59^, ^99^ thus supporting a possible mechanistic basis for associating frailty with endothelial dysfunction.

## 5 THE RhoA/RHO KINASE PATHWAY

**FIGURE 2:**
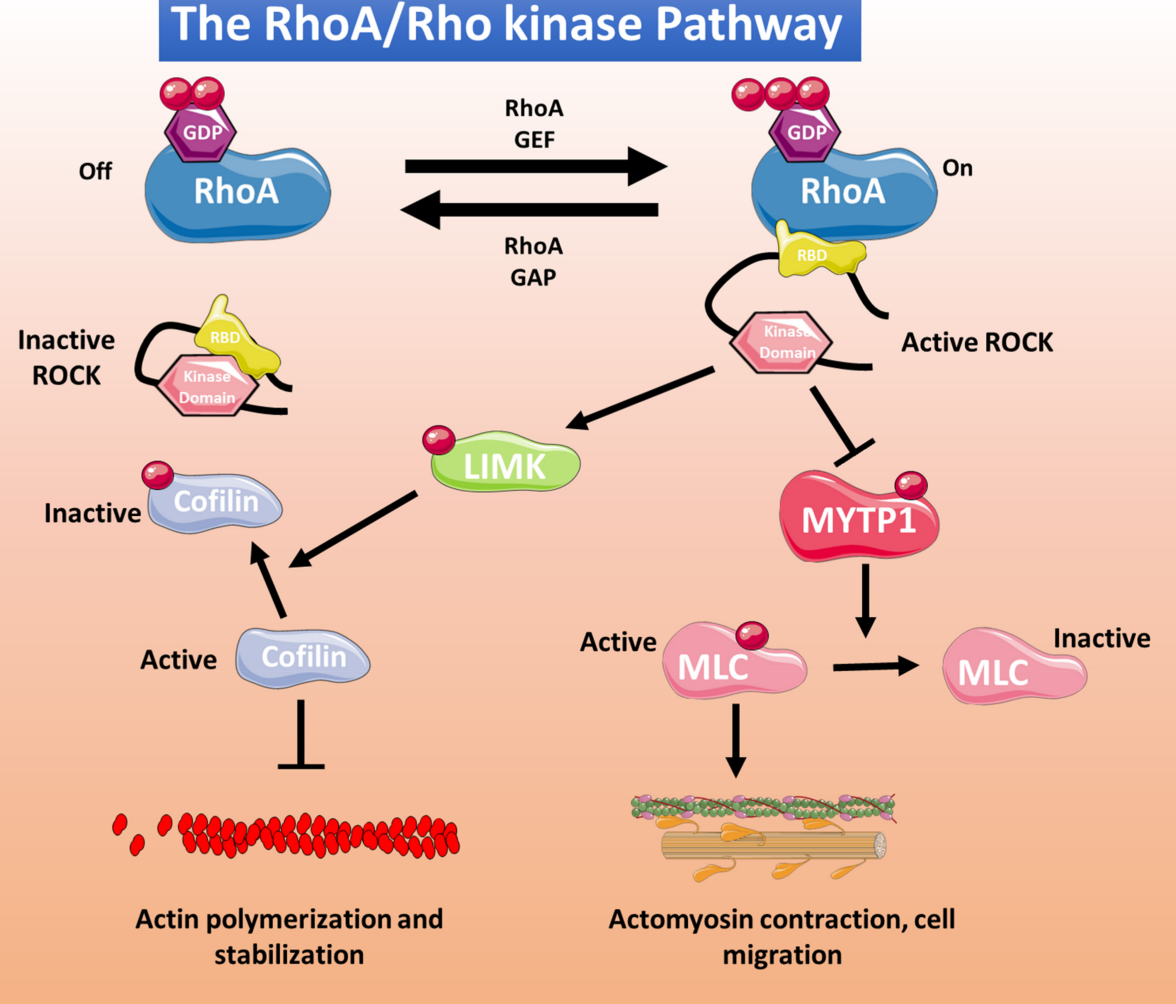
Classical RhoA/Rho kinase pathway. Different stimuli can induce RhoA activation, through guanosine exchange factors (GEF) or inhibition through GTPase‐activating protein (GAP). Activated RhoA interacts with the Rho‐binding domain (RBD) domain of Rho kinase, releasing and activating the kinase domain. Activated Rho kinase phosphorylates multiple cell targets, including LIM kinase (LMK) and myosin phosphatase target subunit 1 (MYTP1). Phosphorylated LIMK phosphorylates, and thus inactivates, cofilin, inducing actin polymerization and stabilization. Phosphorylated MYTP1 is inactivated, resulting in the stabilization of the phosphorylated and active form of myosin light chain (MLC), thus promoting actomyosin contraction and cell migration.

The RhoA is one of the best‐known members of a large family of small GTPases that includes Rho, Rac and Cdc42 members. RhoA and its downstream targets and effector proteins, the Rho kinases, play important roles in many cellular functions, particularly cellular cytoskeletal reorganization^100^ (Figure 2). The two known Rho kinase isoforms are ROCK1 (ROCK β) and ROCK 2 (ROCK α), which share 65% sequence homology. Both isoforms are expressed in ECs, with ROCK1 localized in plasma membrane and ROCK2 in the cytoplasm.^101^ In ECs, the RhoA/Rho kinase pathway inhibits NO production,^102^ and excessive pathway activity induces oxidative stress and promotes CVD development.^102^ ROCK1 and ROCK 2 are both upregulated by angiotensin II and interleukin 1β, but whereas ROCK1 is cleaved by caspase 3 (an important step in erythroblast development), ROCK2 is cleaved by granzyme B released by cytotoxic lymphocytes, basophils, mast cells and vascular smooth muscle cells (VSMCs), implicating thus the inflammatory action of granzyme B in the activation of ROCK2. Also, ROCK2 is the main Rho kinase isoform in cells of the cardiovascular system.^102^ Previous work from our group has shown that abnormal RhoA/Rho kinase pathway activation contributes to multiple pathological processes associated with thrombotic complications, such as metabolic syndrome,^13^ inflammatory bowel disease^103^ and cocaine‐related cardiovascular pathology.^104^

## 6 THE RhoA/RHO KINASE PATHWAY IN AGE‐RELATED FEATURES OF VASCULAR DYSFUNCTION

### 6.1 RhoA/Rho kinase in endothelial dysfunction and prothrombotic conditions

The RhoA/Rho kinase pathway is important for normal endothelial homeostasis, although studies with endothelial‐specific RhoA knockouts mice demonstrate that its lack of function can be compensated during the embryonic development.^11^ Nevertheless, abnormal activity can lead to EC dysfunction.^105^ RhoA/Rho kinase signalling plays a pivotal mechanosensory role in actin dynamics in ECs, promoting cell contraction and endothelial sensitivity.^106^ Moreover, it has been proposed that the basal activity of RhoA/Rho kinase signalling mediates normal intrinsic barrier‐protective activity at EC margins, but abnormal activation by vasoactive agents (as thrombin) can disrupt this barrier, facilitating the breakdown of intercellular junctions and increasing endothelial permeability.^107^

The RhoA/Rho kinase pathway is an important suppressor of endothelial NO synthase (eNOS) and increases oxidative stress.^108^, ^109^ RhoA/Rho kinase activation also upregulates the proapoptotic protein Bax through the tumour suppressor protein p53 to induce a mitochondrial death pathway.^110^ There is evidence that RhoA/Rho kinase pathway activation by PKC underlies the increase in arginase I expression and activity induced by oxidative stress in bovine aortic ECs, related to a decrease in NO production due to the competition with eNOS for the substrate arginine.^111^

The RhoA/Rho kinase pathway is involved in endothelial microvasculature damage caused by lipopolysaccharide (LPS). The anti‐inflammatory effect of catalpol, the major active compound in *Rehmannia glutinosa*, protects against LPS‐induced blood–brain barrier disruption by decreasing RhoA and ROCK2 mRNA and protein expression, reversing LPS‐induced cytoskeletal actin disaggregation in brain microvascular mouse ECs.^112^ In rat pulmonary microvascular ECs, the pro‐apoptotic effect of LPS appears to be mediated by Rho/Rho kinase with JNK and p38 MAPKs as downstream effectors, since the ROCK inhibitor fasudil blocked JNK and p38 activation and the appearance of apoptosis markers.^113^

The RhoA/Rho kinase pathway is also involved in impaired angiogenesis and focal adhesion dysregulation.^105^, ^114^ Angiogenesis is essential for physiological vascular function and recovery from ischemic conditions. In fact, there is a relationship between endothelial dysfunction and impaired NO production with angiogenic impairment, which contributes to age‐related decline in microvascular density, decreased myocardial blood supply, impaired capacity to adapt at hypoxia, and exacerbated ischemic tissue injury.^83^ Thus, impaired angiogenesis is closely related to endothelial dysfunction and CVD. Endothelial homeostasis is crucially regulated by the Gα‐coupled heptahelical thromboxane A2 receptor, and the thromboxane A2 receptor/Gα13/RhoA/C/Rho kinase/LIMK2 pathway inhibits VEGF‐mediated human umbilical vein EC sprouting and promotes EC tension and focal adhesion dysregulation.^114^

The RhoA activity exerts an inhibitory effect on the angiogenic capacity. The expression of constitutively active RhoA (G14V/Q63L) in HUVEC inhibits endothelial proliferation, migration, tube formation and in vitro angiogenic sprouting, which I abrogated with a non‐active dominant‐negative version of RhoA (T19N). However, this induction of endothelial dysfunction and antiangiogenic effects by active RhoA seems to be independent of its downstream effectors, ROCK and LIMK.^105^ On the other hand, RhoA/ROCK pathway may be involved in pathological angiogenesis. Xueke et al.^115^ showed that the compound erianin inhibits pathological angiogenesis in vitro and neovascularization in vivo in a hypoxia‐induced retinopathy in adults and embryonic zebrafish, by inhibiting collagen binding to α2 and β1 integrins and suppressing the intracellular RhoA/ROCK1 signalling pathway. In the same line, Yoshifumi et al.^116^ reported that ripasudil (ROCK inhibitor) prevented retinal edema, reduced the size of the nonperfusion area and improved retinal blood flow in a murine model of retinal vein occlusion by suppressing retinal phosphorylation of MYPT‐1 and inhibited disorganisation of tight junctions 1 in human retinal microvascular endothelial cells. The increase in vascular resistance and rigidity is associated with vascular stiffness, and when it is deregulated in vascular smooth muscle cells, it is a major cause of cardiovascular disorders.^117^

This range of actions establishes RhoA/Rho kinase pathway activation and dysregulation as a major cause of ageing‐associated vascular dysfunction and suggests that it may be an attractive therapeutic target.^12^ Studies with specific RhoA/Rho kinase inhibitors have shown multiple benefits, for example in the control of blood pressure, decreased cardiac damage in ischemia/reperfusion models, an enhanced vascular antioxidant response and normalization of vascular parameters and NO production in a mouse model of age‐induced endothelial dysfunction.^118^, ^119^, ^120^, ^121^, ^122^, ^123^ In an ex vivo study, Pereira et al. demonstrated an increased circulating endothelial cells and an increased activity of RhoA kinase activity by MYPT1‐P/T phosphorylated in circulating leukocytes from cocaine‐dependent individuals and in aortic cells from cocaine‐treated rats. Atorvastatin and the Rho kinase inhibitor Y‐27632 protect endothelial function in vitro by inhibiting pro‐adhesive and prothrombotic changes induced by cocaine or plasma from chronic cocaine consumers.^104^, ^124^ These data suggest that activation of RhoA/Rho kinase pathway plays a key role in endothelial dysfunction induced by injuries like cocaine consumption and that inhibition of this pathway may provide therapeutic benefits.

### 6.2 RhoA/Rho kinase in increased vasoconstriction and hypertension

The RhoA/Rho kinase activation has been observed in patients with heart failure, a population with high prevalence of frailty (~79%), and the Rho kinase inhibitor fasudil reduces vascular resistance and improves vasodilation.^125^, ^126^ RhoA/Rho kinase activation in arteries has also been shown in mouse models of aging^127^ and has been suggested to contribute to age‐related blood pressure elevation, possibly via greater peripheral vasoconstrictor tone in older adults.^128^ There are also several studies linking RhoA/Rho kinase to both natural and induced senescence in various cell types, including annulus fibrosus cells,^129^ kidney cells,^130^ internal anal sphincter smooth muscle cells,^131^ corpus callosum cells^132^ and mesenteric arterial smooth muscle.^133^

There is also abundant evidence implicating the RhoA/Rho kinase pathway in VSMC hypercontraction, VSMC proliferation and migration in the media, inflammatory cell accumulation in the adventitia, inhibition of NO production and increased oxidative stress.^134^, ^135^, ^136^, ^137^ In another study, the vascular proteome of wild‐type male C57BL/6 mice was analysed by hierarchical clustering to detect proteins showing significant age‐related changes. In this analysis, several proteins associated with the RhoA/Rho kinase pathway showed changes consistent with hypertension and cerebral perfusion dysregulation, suggesting that the RhoA/Rho kinase pathway is an important target for age‐dependent hypertension.^138^

The RhoA/Rho kinase pathway has been linked to age‐dependent VSMC dysfunction, including the age‐associated decrease in contractile function. In soleus muscle feed arteries from aged (24‐month‐old) rats, elevated levels of pROCK1 and pROCK2 were associated with depressed contractile ability, α‐actin stress fibres, recruitment of proteins to cell‐matrix adhesions and an increase in integrin adhesion to the matrix related with increased cell stiffness.^139^ In mouse VSMCs, a non‐canonical Wnt5a/RhoA activation pathway shows a putative association with age‐related salt‐sensitive hypertension by increasing calcium sensitivity triggered by the decline in the protein Klotho with age.^140^ The RhoA/Rho kinase pathway has also been linked to vascular reactivity and dysregulation associated with phenylephrine‐induced vasoconstriction. In aging spontaneously hypertensive rats, ROCK‐2 expression and activity are excessively increased, accompanied by decreased myosin light chain phosphatase (MLCP) activity and an increase in phosphorylated MLC, leading in turn to increased α1‐adrenergic‐induced vasoconstriction.^133^

## 7 CONCLUSIONS

**FIGURE 3:**
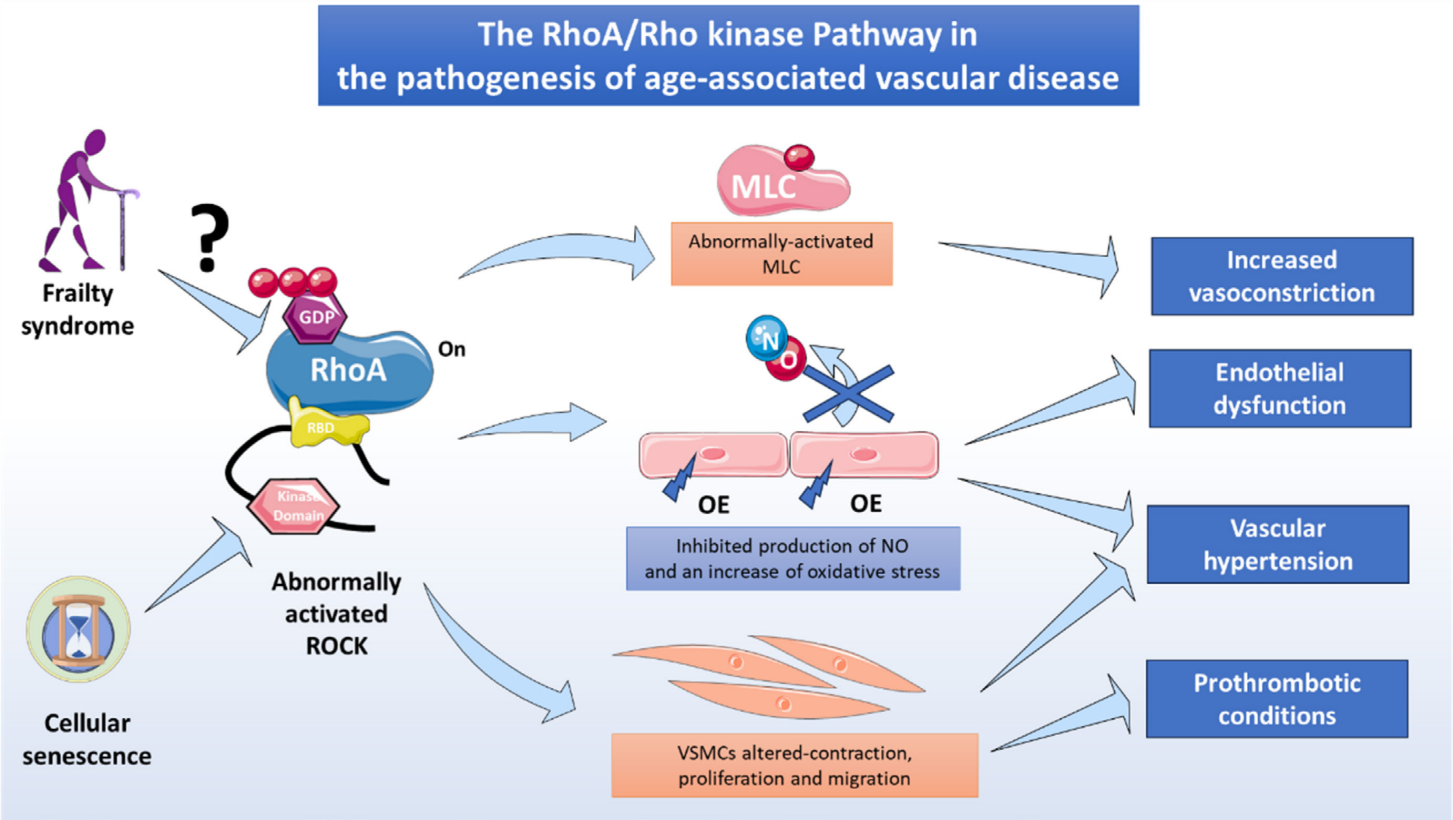
RhoA/Rho kinase pathway activation in age‐related vascular disease. Abnormal activation of the RhoA/Rho kinase pathway has been linked to aging and cellular senescence. Frailty syndrome, directly related with unhealthy ageing, may increase the risk of abnormal RhoA/Rho kinase activation, in turn triggering pathological cell mechanisms that lead to age‐related vascular disease and promote a prothrombotic status in elderly people. MLC, myosin light chain; OE, oxidative stress; VSMCs, vascular smooth muscle cells.

The RhoA/Rho kinase pathway is a crucial signalling component in the vascular system and particularly in ECs and has been extensively studied in diverse pathophysiological settings, including endothelial dysfunction, impaired angiogenesis, inflammation and apoptosis. In recent years, attention has focused on the emerging association between RhoA/Rho kinase signalling components and cell senescence and ageing in diverse cell types. However, further research is needed to define the role of the RhoA/Rho kinase pathway in vascular aging. The findings reviewed here suggest that RhoA/Rho kinase pathway activity in endothelial dysfunction is highly relevant to frailty syndrome and could provide a promising route to the development of therapeutic interventions to prevent vascular aging (Figure 3).

## AUTHOR CONTRIBUTIONS

**Iván Palomo:** Conceptualization (equal); investigation (equal); writing – original draft (equal). **Sergio Wehinger:** Investigation (equal); writing – original draft (equal). **Vicente Andrés:** Conceptualization (equal); writing – review and editing (equal). **Francisco J. García‐García:** Investigation (equal); writing – review and editing (equal). **Eduardo Fuentes:** Conceptualization (equal); writing – original draft (equal); writing – review and editing (equal).

## CONFLICT OF INTEREST STATEMENT

The authors have no conflicts of interest to disclose.

## Data Availability

Data derived from public domain resources.
